# Medical Society of London

**Published:** 1851-06

**Authors:** 


*Medical Society of London.*—Mr. Thomas H. Wakely exhibited a set of *New Instruments for the cure of stricture.* Mr. Wakely said that he should have preferred sending a paper with the instruments, giving a full description of them, and the details of a few cases in which they have been used, but he had been informed that the "list of papers" for the remainder of the session was full. He therefore availed himself of the opportunity thus afforded by the regulations of the Society for submitting the instruments to their consideration at that time. He remarked, that the subject of the treatment of stricture of urethra had been much discussed within the last year or two, and had given rise to a great deal of controversy. It certainly was not a settled question what should be done in cases of severe permanent stricture. Mr. Syme, the distinguished surgeon of Edinburgh, had, as was known, recommended, where the ordinary means of treatment had failed, the division of such strictures by perinæal section. Probably, the instruments which he then had the honor of bringing before the notice of the Society would, in some cases at least, render such an operation unnecessary. He had used them in several cases already with very satisfactory results. The instruments he at first used were by no means of a refined or perfect manufacture, yet the advantages obtained were of a decided character. The instruments he now produced had been manufactured by Messrs. Weiss & Co., and, as might be expected, they were very perfectly executed. They consist of—

1. A catheter, thirteen inches in length, of a very small size, slightly curved at the extremity; the stem quite straight, and having at the end a worm, for the reception of the screw of the directing rod.

2. A small thumb-slide, (removable at pleasure,) screwing closely upon, and acting as a handle to, the catheter.

3. A steel rod, which passes into the catheter as far as the screw, at which part both are united by two or three turns of the rod. The rod makes an addition of five inches to the length of the catheter. The rod and catheter combined form the index-rod, or director, for the metallic and elastic tubes.

4. Of the silver straight tubes, there are nine of graduated sizes; the first is only one size larger than the index-rod, and the others regularly increase in circumference; the last, or No. 10, corresponding with that number of the ordinary bougie. These tubes are all of a conical shape at there distal extremities, and are so constructed as to fit the mouth of each tube with extreme exactness at the surface of the index-rod. They thus slide with the most perfect ease along that guide, and being directed by it, if the rod be in its proper situation, the tubes cannot take a wrong course or make a false passage, but must pass through the stricture.

5. There are also three *elastic tubes*, composed of a flexible metal, covered with elastic-gum fabrics. This combination gives to the instrument very considerable strength, without rendering it clumsy or bulky. The extremity of each of these flexible tubes has the same form as that of the silver tubes, and fits with perfect accuracy the surface of the index-rod.

Supposing, then, that a patient having stricture of the urethra is before the surgeon for operation, the mode of proceeding is as follows:—

First, introduce the catheter, as gently and with as much care as possible, completely through the contracted part of the urethra into the bladder. Having done this, withdraw the stylet, and the surgeon having satisfied himself, by the escape of urine, that the instrument is in the bladder, insert the smaller extremity of the steel rod into the catheter, and having secured it, by making two or three turns of the rod, remove the thum-slide, and then pass No. 3 silver tube upon the index-rod right through the stricture or strictures. In performing this operation, the passage of the instrument will be much facilitated by giving to the flanges a rotatory motion as they are held between the fingers and thumb. This tube being withdrawn, the others may all be passed in a similar manner, and in regular succession. The number to be introduced must of course be determined by the operator. After the last metallic tube is withdrawn, an important object is still to be secured—that of *keeping the command of a free urethra.* How is that to be done? This certainly is a point of considerable importance. Mr. Wakely stated that he was happy to say that it might be accomplished with the greatest ease, by passing of the *elastic tubes* over the index-rod, as done in the case of the silver tubes. One of the flexible tubes being now in the bladder, *the index-rod is to be withdrawn through it;* this may be done with the most perfect ease and facility. The *flexible tube* may be left in the bladder, to serve the purpose of a catheter, and also to afford a safe channel or guide for the re-introduction of the silver catheter or index-rod.

The Society would not fail to perceive that the action of these instruments was safe and simple, and he had the pleasure of stating that the use of them had given him very great satisfaction. The application of the knife for the relief of strictures had been much condemned, although it had been strongly advocated by Mr. Syme, who was undoubtedly a high authority. Still he (Mr. Wakely) could not refrain from expressing a belief that there were strictures which might be removed by the instruments now before him, although Mr. Syme might consider that in such cases the perinæal section would be absolutely necessary to effeet a cure. Time and experience in the trial of both plans would be required to enable a decision to be formed as to their merits. Mr. Syme had remarked, in a letter published that day, that he had endeavored to establish two positions: "First, that the division of a stricture by external incision, upon a grooved director, passed fairly through the contracted part, is an operation free from all ordinary sources of danger. Secondly: that by this procedure, strictures which resist every other mode of treatment, and are apt to resent seriously even the gentlest use of simple bougies, may be speedily removed, so as to allow instruments of the largest size to be introduced without difficulty or inconvenience." The first proposition demanded particular attention, because he thought that the plan of treatment now proposed, by tubular expansion, would in many cases of stricture contemplated by Mr. Syme, render the perinæal section unnecessary. If the grooved director mentioned by Mr. Syme could be "passed fairly through the contracted part," of course the small-sized catheter or index-rod now shown could also be guided through the stricture into the bladder. Necessarily, if the one instrument could be passed, so could the other; and the passage being thus secured, the tubes, both metalic and flexible, might be made to take the same course, without the slightest danger of making a false passage. In some very obstinate and inveterate strictures he had succeeded in affording relief, almost without difficulty. Some of the strictures appeared to be of the worst possible form. He was glad to perceive some gentlemen in the room who had been present when the instruments were used at the hospital. Amongst them he observed his colleague Mr. Gay, who could acquaint the Society with the result of the treatment as pursued in the case of one of his patients In that instance the man had been treated in the ordinary way, but without success. It was suggested that it was a case which would effectually test the efficiency of the new treatment. The rod and the tubes were introduced in the presence of Mr. Guthrie and several other gentlemen. After Mr. Gay had very cleverly, but not without some difficulty, introduced a No. 2 catheter, the metallic tubes from No. 3 to No. 9 were passed without a check. No. 8 elastic tube was then passed on the directing catheter, and the latter instrument withdrawn, leaving the elastic tube in the bladder. He might appeal to Mr. Gay as to the accuracy of the statement. Mr. Wakely believed that in the hands of others the effects produced by the instruments would prove as satisfactory as they had been to himself. At the hospital with which he was connected, the opportunities for proving their utility were very frequent, and it would afford him pleasure to show any practitioner who might honor that institution with a visit, the manner in which they were employed. The instruments had been seen by Sir B. Brodie, Mr. Guthrie, Mr. Stanly, Mr. Fergusson, and other distinguished surgeons, who all approved of the principles of treatment which their use involved. In placing the instruments before the Society and the proffession, he felt confident that they would receive a fair and candid trial. On a future oocasion he should take an opportunity of offering to the notice of the Society the further result of the treatment.

